# The Link Between Periodontal Hygiene Status and Pregnancy: Awareness and Understanding Among Iraqi Pregnant Women

**DOI:** 10.1155/ijod/8889305

**Published:** 2025-06-16

**Authors:** Athraa Ali Mahmood, Banaz Jabbar Ali, Ban Karem Hassan

**Affiliations:** Department of Periodontics, College of Dentistry, Mustansiriyah University, Baghdad, Iraq

**Keywords:** awareness, gingivitis, oral knowledge, periodontal hygiene, pregnancy

## Abstract

**Objective:** To evaluate oral health knowledge and habits, as well as awareness of periodontal hygiene, and its impact on pregnancy outcomes among Iraqi pregnant women.

**Material and Methods:** An observational cross-sectional study involved a survey of 387 pregnant women in Baghdad city. To gauge their level of awareness and knowledge, a pretested, closed-ended questionnaire with nine items about oral health was given out.

**Results:** A significant proportion of pregnant women surveyed appeared unaware of developing gingivitis during pregnancy. Regarding knowledge about the causes of pregnancy gingivitis, nearly half of the surveyed pregnant women (46.9%) were not aware of its origins. A majority of the surveyed pregnant women (72.7%) were unaware of the potential adverse pregnancy outcomes related to pregnant gingivitis. In addition, there was a significant difference in knowledge scores when comparing pregnant women based on their educational levels, and more than half of the women (56.25%) did not undergo their dental checkups during pregnancy. A substantial 82.7% of the surveyed pregnant women believed in the importance of receiving dental health education at the onset of pregnancy.

**Conclusion:** The findings of this investigation supported the need for interaction between dental practitioners and gynecologists, including routine dental checkups during antenatal visits of pregnant women, as essential to develop and improve the habits of awareness of the need for periodontal hygiene during pregnancy. Furthermore, there was a significant association between educational level and awareness and knowledge among pregnant women about the relationship between periodontal health and pregnancy.

## 1. Introduction

Periodontal disease is a critical chronic inflammatory response of the gingival tissue and its supporting structures. It ranges from gingivitis, which is a superficial and reversible affection of the gingiva and periodontitis, characterized by the destruction of connective tissue and dental bone support tissues that can lead to tooth loss. It has been demonstrated to produce systemic infection in different pathways “by shared risk factors (microbial toxins); periodontium acting as a reservoir of inflammatory mediators; and the subgingival biofilms acting as reservoirs of pathogenic bacteria,” that impairs general health and can lead to several systemic illnesses, including cardiovascular problems, diabetes mellitus, and hypertension [1, 2]. In recent times, different studies have demonstrated that periodontal disease may contribute to adverse pregnancy outcomes, such as premature delivery, low birth weight babies, preeclampsia, miscarriage, or early pregnancy loss [3, 4].

Pregnancy is a unique stage for women, characterized by numerous physiological and emotional changes. Pregnant women have been documented to have a variety of diseases in the oral cavity [5]. The exaggerated inflammatory response of the periodontium to bacterial plaque has been attributed to the increased secretion of gestational hormones (mainly estrogen and progesterone) during pregnancy [6].

One of the most important steps in promoting oral health is educating the public about dental care, which is essential for overall quality of life [7]. Maintaining good oral hygiene throughout an induced perinatal period can help lower the amount of oral bacteria and inflammatory mediators that transfer to the embryo or fetus through the placenta with the blood [8]. Moreover, pregnant women and those considering pregnancy should receive a periodontal evaluation and appropriate treatment, according to the “American Academy of Periodontology” [9, 10].

Personal knowledge, along with proper daily tooth brushing and plaque control, can reduce the progression of oral diseases, including periodontal disease and dental caries [11]. In addition, the objective of regular prenatal care is to guarantee the good health of the mother and the subsequent delivery of healthy children [12]. However, even in cases where they exhibit indications of oral disease, a significant number of pregnant women choose not to pursue or obtain any dental care due to low socioeconomic status, low educational achievement, and awareness of the importance of periodontal and oral health [13–15]. Therefore, the current study's goal was to assess participants' knowledge and awareness of the association between periodontal hygiene status and pregnancy outcomes among Iraqi pregnant women. The outcomes would provide a baseline for developing a program of oral health education aimed at enhancing the oral health of Iraqi pregnant patients receiving medical care.

## 2. Materials and Methods

A randomly selected sample of 387 pregnant women was included, aged from 18 to 50 years, who visited government maternity centers in central Baghdad and private clinics in Baghdad between May 2021 and August 2023. The sample size was determined using the G^*∗*^Power 3.1 software, and a power analysis was performed for both the chi-square test and one-way analysis of variance (ANOVA) to assess the responses across five educational-level groups. For ANOVA, a sample of 324 participants was required, assuming a medium effect size *f* = 0.25, a significance level (*α*) of 0.05, and a power of 0.80. For the chi-square test, a sample of 240 individuals was calculated based on similar statistical parameters. A larger sample size of 387 participants was randomly selected to ensure adequate statistical power.

The study questionnaire was revised from a previously published survey [16] done on Saudi pregnant women to assess their level of knowledge and perception regarding pregnancy gingivitis, with minor modifications to bring it into line with the local situation, and all questions were translated into Arabic. The reliability of the questionnaire was assessed using Cohen's kappa test, which yielded a kappa value of 0.81, indicating very good agreement. Before data collection, the questionnaire was pretested on a small sample of 30 pregnant women to evaluate its clarity and relevance. Additionally, expert review by periodontists confirmed the appropriateness and validity of the questions.

The questionnaire consisted of nine questions assessing participants' knowledge and attitudes regarding oral hygiene and dental care habits during pregnancy. The first section (questions [1]–[4]) gathered demographic information, including age, educational status, occupation, number of pregnancies, and trimester. Participants were informed about the study's purpose, and informed consent was obtained before participation. Those who declined to participate were excluded from the study. The survey ensured anonymity, as no personal identifiers (e.g., names) were collected. For illiterate participants, each question was read aloud, and the available answer choices were explained.

To quantify participants' knowledge, a “dental knowledge score” was established. One point was assigned for each correct or acceptable response, while “Do not know” responses and unanswered questions were excluded from scoring. The final knowledge score ranged from 0 to 7, with higher scores indicating greater awareness. Two questions, (Getting a dental checkup done during or before pregnancy?) and (Do you believe that dental health education should begin at the onset of pregnancy?) were excluded from the scoring system.

The protocol was approved by the College of Dentistry, Al-Mustansiriyah University, in compliance with institutional ethical guidelines.

The data was entered in Microsoft Excel and put to statistical analysis using IBM-SPSS Statistics 26 software. The data were described as frequency, percentage, mean, standard deviation, and so on for descriptive statistics. The Shapiro–Wilk test assessed the data, which revealed that the data were normally distributed. Consequently, comparisons between the educational level groups were analyzed by one-way ANOVA, and the chi-squared tests were used to find the association between oral hygiene awareness and educational levels. The graph was created with the GraphPad Prism program version 10.

## 3. Results

The demographic data in Table 1 show that participants had a mean age of 27.7 (±7.6), and half were in the age group (25–35). Concerning educational background, 26.8% of participants had primary education, 26.8% had secondary education, 10.2% completed higher secondary education, 29.1% joined university, and only 1.6% held a postgraduate. A substantial majority (78.7%) of participants were recognized as housewives, while the remaining were employed.

Furthermore, 45.9% of participants informed having two to three children. Regarding pregnancy status, nearly half of the participants were in the third trimester, and 39.3% were in the second trimester.

Table 2 shows that a significant number of the pregnant women surveyed lacked awareness about developing gingivitis during pregnancy; 52.8% had not heard about it, and only 35.1% were aware. At the same time, 12.1% had heard about it but were not fully aware. Participants with 67.6% said they did not exhibit any signs of pregnancy gingivitis. Regarding knowledge about the causes of gingivitis during pregnancy, nearly half of the pregnant women (46.9%) were not aware of its origins, and 42.9% believed that daily tooth brushing along with flossing could prevent gingivitis in pregnant women. In comparison, 32.2% did not know about it. 61.5% of pregnant women believed that dentists would rather treat pregnant gingivitis with drugs. 40.7% of pregnant women lacked information about the effects of pregnancy on gingivitis. A majority of pregnant women (72.7%) did not know of the potential adverse outcomes of pregnancy related to pregnant gingivitis.

**Figure 1:**
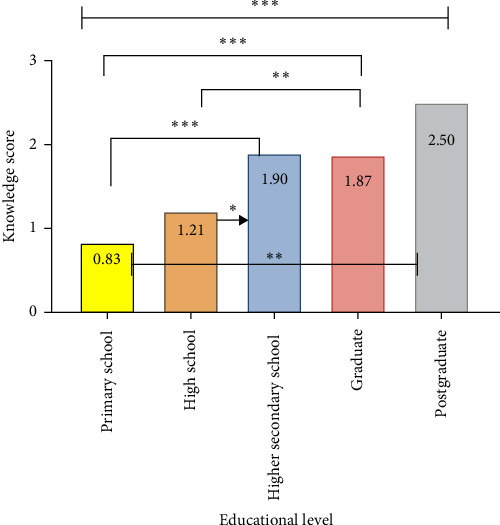
Evaluation of knowledge scores across different educational level groups using one-way ANOVA and Bonferroni post hoc test. *⁣*^*∗*^*p*  < 0.05, *⁣*^*∗∗*^*p*  < 0.01, and *⁣*^*∗∗∗*^*p*  < 0.001.

There was a significant association between all the responses of pregnant women and the level of education. An ANOVA test shows a significant difference in knowledge scores when comparing the responses of pregnant women based on their educational levels, as in Figure 1.

**Figure 2:**
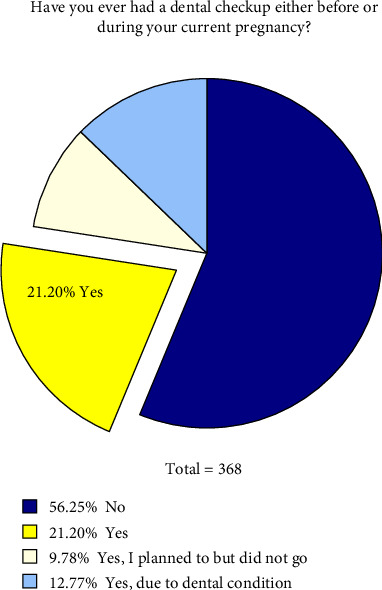
Frequency distribution of response on dental checkups before and during pregnancy.

**Figure 3:**
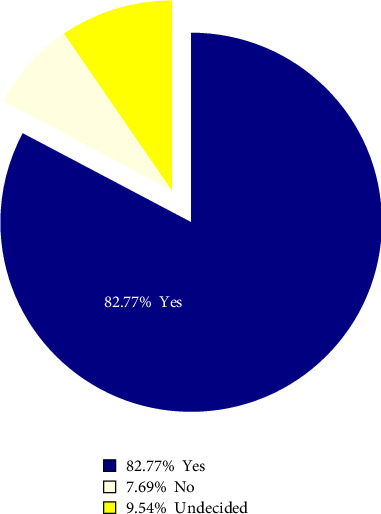
Frequency distribution of response to the importance of dental health education at the onset of pregnancy.

Figure 2 reveals that more than half of the women (56.25%) did not undergo dental checkups during pregnancy (Figure 3). A majority of pregnant women (82.7%) believe that dental health education should begin at the onset of pregnancy.

## 4. Discussion

Oral health is an important component of overall health, and its importance grows during pregnancy, especially in light of emerging evidence that low fetal weight, as determined by ultrasound screening, is linked to deterioration of the periodontal health in pregnant women [17]. Pregnancy causes complex physical and hormonal changes that affect practically every organ system. These changes also have an impact on the oral cavity. Gingivitis and periodontal infection are the most common oral complications connected with pregnancy [18].

Gingivitis is the most frequent periodontal disease during pregnancy, with incidence rates ranging from 30% to 100%, according to studies [19]. Pregnancy-related gingival inflammation is induced by dental plaque and aggravated by endogenous steroid hormones [20]. During pregnancy, there is a rise in estrogen metabolism by the gingiva and prostaglandins, which causes changes in the gingival tissues [21]. The immune system weakens due to changes in sex hormone levels, affecting the pace of collagen production; as a result, the body is unable to heal the gingiva [22].

The most prevalent oral disease during pregnancy can be prevented with basic procedures like daily tooth brushing and flossing. Such beneficial behavior, however, would be influenced by an individual's oral health education and attitudes [23].

As a result, there is a need to grasp pregnant women's perspectives and knowledge about oral health; thus, initiatives to promote dental health can be directed toward early detection, treatment, and education regarding pregnant gingivitis and periodontitis, eventually preventing any possible detrimental effects on unborn children. Therefore, the purpose of this study was to provide an overview of pregnant women's oral health knowledge and perspectives, as well as their awareness of pregnancy gingivitis and its impact on pregnancy outcomes in Iraqi health facilities. In this study, we found that only 35.1% of the respondents were fully aware of developing pregnancy gingivitis during pregnancy, and this was higher than that found in a study done on pregnant women in Saudi Arabia, which assessed their level of knowledge and perception regarding pregnancy gingivitis, where 25.89% of them were aware of it [16].

In addition, about half (52.8%) of the respondents answered (No, I'm not aware), while the remaining (12.1%) of them were not fully aware but had heard it. From the result, we can see that the level of awareness about the gingivitis in pregnancy is less than optimal, and here comes the role of the dentist in educating pregnant women about the increased prevalence of gingivitis during pregnancy and it creates a space for continual education and the promotion of the health system's informative program. In addition, we can see that as pregnant women's educational level increases, the awareness about pregnancy gingivitis increases. This was consistent with other studies that found that pregnant women with higher levels of education had a much higher level of oral health awareness than those with lower or no education. The reason for this could be attributed to their increased social engagement and subsequent exchange of ideas and viewpoints, raising their oral health knowledge level [24].

When pregnant women inquired if they had any signs of pregnant gingivitis, 15.1% reported they had swollen and reddened gums, 8.2% had bad breath, 5.3% had gum bleeding, and 3.7% said they experienced red spots over the oral lining. While most participants (67.6%) answered that they didn't have any signs of gingivitis, this was higher than what was found in another study [23], in which 13.9% felt that they had no signs of pregnant gingivitis. Although gum inflammation is common during pregnancy, gingivitis is unlikely to occur if dental hygiene is maintained and no food particles or plaque are present because hormones alone are insufficient to trigger inflammation [7]. This could explain the high rate of pregnant women who reported that they don't have any signs of gingivitis. Nonetheless, because this study relied on self-reported data, the results are prone to bias.

When pregnant women were questioned about the potential causes of gingivitis during pregnancy in the current study, a high percentage of respondents (46.9%) answered that they did not know the cause of gingivitis and (19.5%) answered it might be the hormonal changes and that was different from that found in a similar study [16], in which the percent was (32.66% and 31.07%), respectively. The remaining answers of participants were as follows: (8.0%) for traumatic tooth brushing, (12.1%) for poor oral hygiene and malnutrition, and (1.5%) for eating fried food. The results reveal that the knowledge and information about the main cause of periodontal disease in pregnancy and the role of hormonal changes are not sufficient.

Regarding the question about the measures that pregnant women believed could prevent pregnant gingivitis, the majority of them (42.9%) were aware of the fact that daily tooth brushing along with flossing could help prevent gingivitis, and this is higher than Saudi pregnant women (35.05%) [16] and less than Chinese pregnant women (84%) [25]. Also, a small percentage of respondents (4.4%) believed that getting a dental checkup done during the second trimester would avoid gingivitis. Many respondents (32.2%) do not know what could help prevent gingivitis during pregnancy, and only 5.9% of participants thought getting dental checkups before pregnancy could prevent pregnancy gingivitis, while 8.3% of them believed that a balanced diet would be beneficial. Few pregnant women (6.2%) answered that monthly scaling will help prevent pregnancy gingivitis.

Brushing teeth with a manual or motorized toothbrush and flossing has long been regarded as the gold standard for regular plaque removal and gingivitis control [26]. However, appropriate nutrition and a healthy lifestyle also play an important role in the well-being of the expectant mother, including periodontal health [27]. Fruits and veggies are high in micronutrients and antioxidants, which benefit and protect periodontal health. According to research, a balanced and healthy diet provides anti-inflammatory and protective effects on periodontal health [28].

From the result, we can see that only a small percentage knew the importance of regular dental checkups before and during pregnancy and the role of a balanced diet in preventing pregnancy gingivitis, so there is a need to increase the awareness and knowledge of pregnant women about the significance of these aspects in prevention, as well as in early diagnosis and treatment of pregnancy gingivitis. Preventive periodontal care might start early in pregnancy with thorough plaque and calculus removal and the patient's oral hygiene instructions. Women must be taught to brush and floss their teeth to eliminate subgingival plaque, and skilled scaling and prophylaxis should be performed as needed [29].

When pregnant women were asked about the treatment dentists provide for pregnancy gingivitis, according to their knowledge, the highest percentage was for medications (61.5%), followed by the (Do not know) answer, which was 19.4%. The remaining treatment options (Surgical removal of swollen gum, Professional scaling, and extract of the affected tooth) were 3.1%, 3.7%, and 4.8%, respectively. At the same time, no treatment option was selected by 7.6% of the participants.

Dentists are essential in delivering the most appropriate treatment to their pregnant patients. The results show that there are misconceptions about the first-line treatment for pregnancy gingivitis. The majority of participants selected medication as the primary treatment option provided by dentists, rather than oral hygiene instruction and professional dental care. However, the correct approach involves educating the patient about the disease, giving the necessary oral hygiene instructions to maintain a healthy oral cavity, and then performing the required scaling and polishing. Such misconceptions must be clarified to motivate pregnant women to seek dental care when necessary. Previous research has found that scaling and root surface debridement should be performed regardless of the pregnancy trimester to promote good oral health [30, 31].

When the study's pregnant participants were asked about the potential impact of pregnancy gingivitis on oral health, the majority of them (40.7%) did not know, and this is higher than that found in Chinese pregnant women, with the percentage of 15% of the same answer [25]. Only 16.6% of pregnant women responded that pregnancy gingivitis could lead to periodontal disease, which was slightly higher than the response of Saudi pregnant women (13.54%) [16]. Also, in this study, 2.0% of respondents answered that pregnancy gingivitis may cause gum tissue overgrowth. As for (No effect, Tooth decay, Tooth sensitivity, and Loose tooth) answers, the percentage was 40.7%, collectively.

Gingivitis is clinically significant since it is a pre-existing stage of periodontitis, the development of which happens only in long-term gingivitis [32]. From the results, we can see that a small percentage knew that pregnancy gingivitis could lead to periodontal disease, so there is a need for educating pregnant women about the adverse effects of untreated pregnancy gingivitis. Dentists play a very important role in society, therefore, we should make pregnant women aware of the effects of pregnancy gingivitis on oral health and general health, and advise them to have a periodic dental examination along with treatment for any dental or periodontal disease.

According to the result of this study, when pregnant women were asked about the possible adverse effect of pregnancy gingivitis on pregnancy outcome, the answer of almost three-quarters (72.7%) of participants was (Do not Know), and this was higher than the Chinese and Saudi pregnant women, in which the percent was (67% and 55.37%), respectively [16, 25]. On the other hand, (17.1%) of participants said there was no effect, and only (6.4%) of them reported that pregnancy gingivitis could lead to preterm births, which was slightly higher than that found in a previous study [33], which showed that a mere 5.1% of participants thought that there could be a connection between periodontal disease and early labor. The majority of participants (53.2%) in a study on pregnant women in northern Jordan were aware that periodontal disorders may have an impact on the course of a pregnancy [34].

Many adverse pregnancy outcomes have been connected to a pregnant woman's poor oral health, including periodontitis, preterm birth, and low birth weight, according to earlier research [4]. Premature infants are a significant public health issue since birth weight constitutes one of the most significant variables in infant growth, development, and survival [35]. López et al. discovered that women whose teeth were treated with scaling or root planing during pregnancy had a lower incidence of preterm birth [36].

Although prior research has suggested that poor dental health in a pregnant woman can contribute to unfavorable pregnancy outcomes, other research has found no link between the two [37]. As a result, this topic is still being researched. While research is still being conducted to determine a cause-and-effect relationship between oral health and pregnancy outcomes, pregnant women should maintain good oral health to avoid associated complications, as prevention has consistently been better than cure. Also, pregnant women should focus on obtaining and acquiring information on oral health care, as well as learning about the relationship between mothers' oral health and the well-being of their offspring. Dentists and the dental profession must tell patients that neglecting dental health raises the chance of negative pregnancy outcomes and increases the likelihood of developing disorders that may affect the newborn's well-being [38].

In the present study, there was a significant association between the level of education and the awareness and knowledge of pregnant women about the relationship between oral health and pregnancy. Similar results were found in a previous study, which reported that educational level was significantly associated with the knowledge of the importance of dental consultation during pregnancy and with the knowledge of the possible link between pregnancy and periodontal diseases [34]. Other studies have found that patients' dental knowledge increased with their level of education [39, 40].

In addition, the present study revealed a significant difference in knowledge scores when comparing pregnant women based on their educational levels. This resembles what was found by Abiola et al., who stated that when comparing the mean knowledge scores, the respondents' level of education was significantly related to the respondents' oral health knowledge. More educated women appeared to have better knowledge than less educated women [12].

## 5. Conclusion

Maintaining oral health is crucial for women, especially during pregnancy, due to its impact on both maternal health and pregnancy outcomes. Raising awareness and knowledge about these effects can encourage better oral hygiene practices among pregnant women.

Our findings indicate that awareness among pregnant Iraqi women regarding pregnancy gingivitis and its implications is insufficient. Therefore, implementing continuous education programs is essential to enhance their understanding of oral health's role in overall well-being and pregnancy outcomes.

Given the significant association between education level and awareness, future research should explore targeted educational interventions to improve oral health literacy among pregnant women. Additionally, integrating oral health education into prenatal care programs could further reinforce the importance of maintaining good oral hygiene during pregnancy.

## Figures and Tables

**Table 1:** Personal characteristics of the survey's pregnant women.

Variable	Frequency (*n*) (%)
Age group (years)	
Less than 25	126 (34.1%)
25–35	185 (50.1%)
36 years and above	58 (15.7%)
Educational level	
Illiterate	21 (5.5%)
Primary school	102 (26.8%)
High school	102 (26.8%)
Higher secondary school	39 (10.2%)
Graduate	111 (29.1%)
Postgraduate	6 (1.6%)
Occupation	
Housewife	263 (78.7%)
Employe	71 (21.3%)
Number of pregnancies	
Primigravida	79 (21.6%)
Two to three pregnancies	168 (45.9%)
More than three pregnancies	119 (32.5%)
Trimester	
First trimester	57 (16.1%)
Second trimesters	139 (39.3%)
Third trimesters	158 (44.6%)

Abbreviations: *n*, number; %, percent.

**Table 2:** Oral hygiene awareness according to different educational levels.

Questions		Total number	Illiterate women	Primary school high	School higher	Secondary school	Graduate	Postgraduate	*p*-Value
(Q1) Being aware of developing pregnancy gingivitis during pregnancy	Yes, I am fully aware	133 (35.1%)	3 (15%)	13 (12.7%)	24 (23.5%)	22 (56.4%)	66 (60%)	5 (83.3)	*p* ≤ 0.001^*∗∗∗*^
	No, I am not aware	200 (52.8%)	12 (60%)	78 (76.5%)	71 (69.6%)	11 (28.2%)	28 (25.5%)	0	
	Not fully aware, but I heard it	46 (12.1%)	5 (25%)	11 (10.8%)	7 (6.9%)	6 (15.4%)	16 (14.5%)	1 (16.7%)	

(Q2) Do you have any following signs of pregnancy gingivitis?	Swollen and reddened gums	57 (15.1%)	5 (23.8%)	8 (8%)	9 (8.9%)	6 (15.4%)	28 (25.5%)	1 (16.7%)	*p* ≤ 0.001^*∗∗∗*^
	Bad breath	31 (8.2%)	3 (14.3%)	10 (10%)	6 (5.9%)	6 (15.4%)	5 (4.5%)	1 (16.7%)	
	Gum bleeding	20 (5.3%)	2 (9.5%)	2 (2%)	3 (3%)	3 (7.7%)	10 (9.1%)	0	
	Red spot over oral lining	14 (3.7%)	4 (19%)	4 (4%)	2 (2%)	1 (2.6%)	3 (2.7%)	0	
	None	255 (67.6%)	7 (33.3%)	76 (76%)	81 (80.2%)	23 (59%)	64 (58.2%)	4 (66.7%)	

(Q3) What may be the cause of gingivitis during pregnancy?	Hormonal changes	66 (19.5%)	1 (5%)	2 (2.2%)	8 (8.8%)	11 (30.6%)	40 (40.8%)	4 (80%)	*p* ≤ 0.001^*∗∗∗*^
	Traumatic tooth brushing	27 (8.0%)	2 (10%)	4 (4.5%)	8 (8.8%)	4 (11.1%)	9 (9.2%)	0	
	Poor oral hygiene	41 (12.1%)	3 (15%)	5 (5.6%)	11 (12.1%)	7 (19.4%)	15 (15.3%)	0	
	Eating fried food	5 (1.5%)	0	1 (1.1%)	1 (1.1%)	0	3 (3.1%)	0	
	Malnutrition	41 (12.1%)	6 (30%)	13 (14.6%)	9 (9.9%)	4 (11.1%)	9 (9.2%)	0	
	Do not know	159 (46.9%)	8 (40%)	64 (71.9%)	54 (59.3%)	10 (27.8%)	22 (22.4%)	1 (20%)	

(Q4) Measures that you think can prevent Pregnancy gingivitis	Daily tooth brushing and flossing	145 (42.9%)	7 (35%)	30 (31.9%)	39 (44.8%)	17 (44.7%)	47 (50.5%)	5 (83.3%)	*p* ≤ 0.001^*∗∗∗*^
	Balanced diet	28 (8.3%)	5 (25%)	6 (6.4%)	5 (5.7%)	2 (5.3%)	10 (10.8%)	0	
	Dental checkup before pregnancy	20 (5.9%)	0	3 (3.2%)	6 (6.9%)	1 (2.6%)	10 (10.8%)	0	
	Monthly scaling	21 (6.2%)	0	5 (5.3%)	2 (2.3%)	6 (15.8%)	8 (8.6%)	0	
	Dental checkup during the second trimester	15 (4.4%)	1 (5%)	2 (2.1%)	0	2 (5.3%)	9 (9.7%)	1 (16.7%)	
	Do not know	109 (32.2%)	7 (35%)	48 (51.1%)	35 (40.2%)	10 (26.3%)	9 (9.7%)	0	

(Q5) What, as per your knowledge, is the treatment dentists provide for pregnancy gingivitis?	Surgical removal of swollen gum	11 (3.1%)	6 (30%)	3 (3.2%)	0	2 (5.3%)	0	0	*p* ≤ 0.001^*∗∗∗*^
	No treatment needed	27 (7.6%)	3 (15%)	6 (6.4%)	9 (9.7%)	2 (5.3%)	7 (6.7%)	0	
	Professional scaling	13 (3.7%)	0	3 (3.2%)	3 (3.2%)	0	6 (5.7%)	1 (16.7%)	
	Extract the affected tooth	17 (4.8%)	0	6 (6.4%)	5 (5.4%)	2 (5.3%)	4 (3.8%)	0	
	Medications	219 (61.5%)	6 (30%)	48 (51.1%)	64 (68.8%)	26 (68.4%)	71 (67.6%)	4 (66.7%)	
	Do not know	69 (19.4%)	5 (25%)	28 (29.8%)	12 (12.9%)	6 (15.8%)	17 (16.2%)	1 (16.7%)	

(Q6) What effect can pregnancy gingivitis have on your oral health?	Overgrowth of gum tissue	7 (2.0%)	1 (5%)	0	2 (2.2%)	0	2 (2.1%)	2 (40%)	*p* ≤ 0.001^*∗∗∗*^
	No effect	16 (4.7%)	1 (5%)	4 (4.2%)	2 (2.2%)	4 (10.8%)	5 (5.2%)	0	
	Periodontal disease	57 (16.6%)	0	7 (7.3%)	16 (18%)	10 (27%)	24 (24.7%)	0	
	Tooth decay	52 (15.1%)	5 (25%)	15 (15.6%)	7 (7.9%)	8 (21.6%)	16 (16.5%)	1 (20%)	
	Tooth sensitivity	39 (11.3%)	2 (10%)	6 (6.3%)	5 (5.6%)	4 (10.8%)	22 (22.7%)	0	
	Loose tooth	33 (9.6%)	4 (20%)	3 (3.1%)	7 (7.9%)	3 (8.1%)	15 (15.5%)	1 (20%)	
	Do not know	140 (40.7%)	7 (35%)	61 (63.5%)	50 (56.2%)	8 (21.6%)	13 (13.4)	1 (20%)	

(Q7) What can be the adverse pregnancy outcome of pregnancy gingivitis?	Preterm birth	24 (6.4%)	3 (15%0	4 (4%)	10 (10%)	2 (5.3%)	5 (4.5%)	0	0.032*⁣*^*∗*^
	No effect	64 (17.1%)	3 (15%)	10 (10%)	12 (12%)	8 (21.1%)	29 (26.4%)	2 (33.3%)	
	Malformation of bones	3 (0.8%)	0	0	0	0	3 (2.7%)	0	
	Cleft lip/cleft palate	11 (2.9%)	2 (10%)	4 (4%)	1 (1%)	1 (2.6%)	3 (2.7%)	0	
	Do not know	272 (72.7%)	12 (60%)	82 (82%)	77 (77%)	27 (71.1%)	70 (63.6%)	4 (66.7%)	

*Note:* Significant at *⁣*^*∗*^*p* < 0.05, *⁣*^*∗∗*^*p* < 0.01, and *⁣*^*∗∗∗*^*p* < 0.001 using chi-squared test.

Abbreviations: *n*, number; %, percent.

## Data Availability

The data that support the findings of this study are available from the corresponding author upon reasonable request.
